# Active Learning in Biostatistics Education for Medical Students: A Systematic Review and Meta-Analysis

**DOI:** 10.7759/cureus.105637

**Published:** 2026-03-22

**Authors:** Néstor Israel Quinapanta Castro, Jorman F Choez-A, Andres F Orbea

**Affiliations:** 1 Department of Doctoral Studies, Faculty of Philosophy and Letters, Universidad de Buenos Aires (UBA), Buenos Aires, ARG; 2 Department of Research, Universidad Regional Autónoma de los Andes (UNIANDES), Ambato, ECU; 3 Methodology of Health Sciences Research, Universidad Internacional de la Rioja (UNIR), Logroño, ESP; 4 Department of Medicine, Universidad Regional Autónoma de los Andes (UNIANDES), Ambato, ECU

**Keywords:** active learning methods, active teaching-learning, epidemiology and biostatistics, medical eduation, teaching biostatistics

## Abstract

Biostatistics is an essential part of medical training and evidence-based decision-making. However, many students find it difficult, which can lead to anxiety and poor performance. Hence, this review aimed to evaluate the effectiveness of active learning methodologies compared to traditional teaching methods for undergraduate medical students. A systematic review with meta-analysis was conducted in accordance with the Preferred Reporting Items for Systematic Reviews and Meta-Analyses (PRISMA) 2020 guidelines. The study protocol was also registered with the International Prospective Register of Systematic Reviews (PROSPERO) under registration number CRD420261299740. Studies on active methodologies for teaching biostatistics to undergraduate medical students were searched in PubMed/MEDLINE, Web of Science, and Google Scholar (2000-2026). Two reviewers performed study selection and data extraction. The primary outcome was academic performance, and the secondary outcomes were satisfaction and perceived knowledge. Risk of bias was assessed using Risk of Bias 2.0 (RoB-2) and Risk of Bias in Non-randomized Studies of Interventions (ROBINS-I). The meta-analysis used the standardized mean difference (SMD) with a 95% confidence interval (CI), a random effects model, and assessed heterogeneity with I² and Egger's test. A p-value of less than 0.05 was considered significant.

A total of 17 studies involving 3,702 medical students were included in the analysis. Active methodologies were found to significantly improve academic performance compared to traditional teaching methods (SMD = 0.83; 95% CI: 0.38-1.27; p = 0.0003; I² = 95%). The effect was also significant by region: America (SMD = 0.51; p = 0.02), Asia (SMD = 1.12; p = 0.02), and Europe (SMD = 0.63; p = 0.0002). In pre-post intervention studies, significant improvements were observed after the intervention (SMD = 2.11; p < 0.0001; I² = 98%). Additionally, higher perceived knowledge (SMD = 0.64; p = 0.009) and student satisfaction (SMD = 0.85; p < 0.00001; I² = 0%) were observed in the intervention group. No evidence of publication bias was detected using Egger's test (p = 0.335). However, several studies presented a moderate to serious risk of bias, and hence, the results should be interpreted with caution. In conclusion, active learning methodologies are effective in improving biostatistics learning.

## Introduction and background

Understanding statistics is crucial for interpreting medical literature, since research findings must be evaluated in light of clinical factors, patient characteristics, and study design. Biostatistics allows researchers and clinicians to determine whether observed results are due to chance or represent true effects [1]. It therefore plays a key role in healthcare decision-making, scientific research, and technological development [2]. For this reason, statistical training is a critical part of medical education and must be strengthened in the curriculum to enhance professional competence and improve the quality of scientific research [3]. Despite its importance, many students, particularly those enrolled in medical programmes, question the usefulness of statistics and report limited interest in numerical subjects [2]. Furthermore, statistics are often perceived as difficult [4], which can lead to anxiety and negative attitudes towards the subject [5]. These perceptions can ultimately result in lower academic performance and difficulties in interpreting scientific articles or conducting research [2].

In this context, several pedagogical approaches have been used in the teaching of health statistics to medical students, including lecture-based learning (LBL), case-based learning (CBL), flipped classrooms, problem-based learning (PBL), and team-based learning (TBL) [6,7]. Among these, active learning has emerged as a particularly impactful strategy, as it fosters engagement and encourages students to take responsibility for their own learning. Unlike traditional passive teaching methods, it enables students to analyze, evaluate, and integrate ideas, thereby more effectively preparing them for professional practice [8]. In higher education, it is based on a constructivist approach that places students at the center of their own learning. This approach promotes participation, reflection, and autonomy, shifting away from passive listening. Various strategies are employed within this approach, including problem-based learning, case studies, projects, collaborative work, and the use of virtual environments [9].

In this regard, it is necessary to gather empirical evidence to assess the impact of these methodologies on the teaching and learning of biostatistics. Previous studies have shown that active learning approaches are beneficial across disciplines. For instance, a meta-analysis of 104 studies in the humanities and social sciences found that active learning significantly enhances student performance compared with traditional lecture-based instruction, with an average improvement of 0.49 standard deviations (SD) [10]. This study aims to assess the effectiveness of active learning methodologies for teaching biostatistics to undergraduate medical students, by comparing them with traditional lecture-based approaches and baseline conditions.

## Review

Methods

This systematic review with meta-analysis was conducted in accordance with the Preferred Reporting Items for Systematic Reviews and Meta-Analyses (PRISMA) 2020 guidelines. The study protocol was also registered with the International Prospective Register of Systematic Reviews (PROSPERO) under registration number CRD420261299740. 

Sources of Information and Search

A systematic search was conducted on MEDLINE/PubMed and Web of Science, supplemented by a review of registers and Google Scholar. Studies published in English or Spanish between 2000 and February 2026 were included. The search strategy, based on the PICO question, combined controlled terms and related keywords: ((("Students, Medical"[MeSH Terms] OR "Education, Medical, Undergraduate"[MeSH Terms] OR "Medical Student"[tiab] OR "Medical Education"[tiab] OR "Undergraduate Medical Education"[tiab])) AND ("Teaching"[MeSH Terms] OR "Education"[MeSH Terms] OR "Curriculum"[MeSH Terms] OR "Teaching"[tiab] OR "Instruction"[tiab] OR "Learning"[tiab]) AND ("Problem-Based Learning"[MeSH Terms] OR "Active Learning"[MeSH Terms] OR "Flipped Classroom"[tiab] OR "Blended Learning"[tiab] OR "Team-Based Learning"[tiab] OR "Case-Based Learning"[tiab] OR "Problem-Based Learning"[tiab] OR "Active Learning"[tiab] OR "Interactive Learning"[tiab]) AND ("Biometry"[MeSH Terms] OR "Statistics as Topic"[MeSH Terms] OR "Biostatistics"[tiab] OR "Statistics"[tiab] OR "Medical Statistics"[tiab] OR "Health Statistics"[tiab])) AND ("2000/01/01"[PDAT] : "2026/02/28"[PDAT]) AND (English[lang] OR Spanish[lang]).

Eligibility Criteria

The eligibility criteria are summarized in Table 1. The studies involved undergraduate medical students who received instruction in biostatistics and assessed active learning methods (problem-based learning, the flipped classroom approach, blended learning, or technology-supported instruction) in a medical school setting. The following quantitative study designs were included: randomized controlled trials, quasi-experimental studies, cohort studies, and analytical observational studies, with traditional teaching or pre-post comparisons as reference groups. Studies involving postgraduate learners, non-medical disciplines, lecture-only interventions, qualitative-only designs, reviews, editorials, protocols, or non-academic contexts were excluded from the analysis.

**Table 1:** Inclusion and exclusion criteria

Category	Inclusion	Exclusion
Population	Undergraduate medical students in preclinical or clinical phases receiving instruction in biostatistics or medical statistics	Postgraduate students or healthcare professionals; students from non-medical disciplines; secondary school or pre-university populations; populations not clearly identified as undergraduate medical students
Intervention	Active learning methods for teaching biostatistics (e.g., problem-based learning, case-based learning, flipped classroom, blended learning, interactive seminars, or use of statistical software)	Traditional lecture-only teaching without active components; self-study without interaction; interventions not focused on biostatistics; interventions targeting non-medical disciplines
Comparator	Traditional teaching methods or within-group pre–post comparisons	Studies without a clear comparison condition or without evaluation of the intervention effect
Study design	Quantitative studies such as randomized controlled trials, quasi-experimental studies, comparative cohort studies, and analytical observational studies	Qualitative studies, reviews, editorials, protocols, opinion papers, or case reports
Context	Undergraduate medical education settings within medical schools, in any country or delivery mode (face-to-face, online, or hybrid)	Studies conducted outside medical schools or in continuing professional education without undergraduate medical students

Primary and Secondary Outcomes

The primary outcome was the objective knowledge of biostatistics score after the intervention. Changes in scores between the pre- and post-tests were also evaluated. The secondary outcomes were student satisfaction, perceived knowledge, and self-reported mastery of biostatistics.

Study Selection Process

The identified records were exported to a bibliographic manager, and duplicates were removed. Two independent reviewers performed the initial screening of titles and abstracts. Potentially eligible articles were then evaluated in full text to determine their final inclusion. Discrepancies were resolved through discussion and consensus. The selection process was documented using a PRISMA flow diagram.

Data Extraction

Data extraction was conducted independently by two reviewers using a standardized, pre-piloted form. Data were gathered on study characteristics (year, country, design), population characteristics (academic year, sample size), a detailed description of the intervention and comparator, assessment instruments, duration of follow-up, and relevant quantitative outcomes. When information was missing or ambiguous, the corresponding authors were contacted to request additional data. Discrepancies in extraction were addressed by consensus.

Assessment of Certainty of Evidence and Risk of Bias

The risk of bias was independently assessed by two reviewers using Risk of Bias 2.0 (RoB-2) for randomized trials and Risk of Bias in Non-randomized Studies of Interventions (ROBINS-I) for non-randomized studies. The overall certainty of the evidence was then evaluated using the Grading of Recommendations, Assessment, Development and Evaluation (GRADE) approach.

Statistical Analysis

Data were analyzed using Review Manager (RevMan) version 5.4. The standardized mean difference (SMD) with 95% confidence intervals (CI) was used as the effect measure. Due to methodological and educational heterogeneity, a random effects model was applied. Statistical heterogeneity was assessed using the I² statistic. A p-value of less than 0.05 was considered statistically significant. Egger's test was used to assess publication bias.

Results

**Figure 1:**
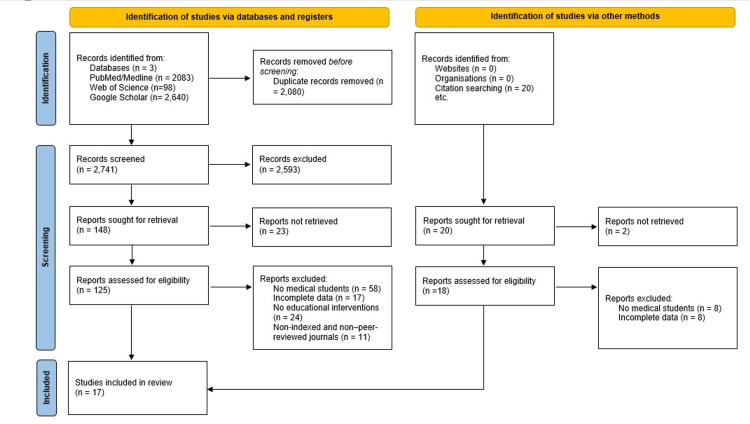
PRISMA flowchart depicting the selection of studies PRISMA: Preferred Reporting Items for Systematic Reviews and Meta-Analyses

A total of 4,841 records were identified (Figure 1). After removing duplicates, 2,741 studies were screened. Seventeen studies met the inclusion criteria and were included in the systematic review, which covered a total sample of 3,702 medical students.

In terms of relative frequency by continent, the largest share of studies originated from Asia (8/17), followed by the Americas (6/17) and Europe (3/17), as shown in Table 2. The studies utilized quasi-experimental designs, randomized clinical trials, and intervention studies without a control group.

**Table 2:** Baseline characteristics of the included studies ^*^Not specified; ^**^Historical cohort comparison PBL: problem-based learning; QE: Quasi-experimental; UPPS: uncontrolled pre–post study; RTC: randomized controlled trial

Study	Country	Design	Sample (n)	Intervention	Duration	Comparator	Main outcomes
Marantz et al., 2003 [11]	USA	QE^**^	862	Case-based discussion (1997–2001 cohorts)	^*^	Lecture-based course (before 1997)	↑ Statistical learning, study design, reasoning, satisfaction (p < 0.01)
Milic et al., 2016 [12]	Serbia	QE	440	Blended learning (Moodle-based)	38 hours	Traditional face-to-face instruction	↑ Final scores (d = 0.43), ↓ Dropout
Quinapanta et al., 2026 [13]	Ecuador	Prospective observational comparative study	47	“3-in-1” active method (article analysis, SPSS simulation, lecture)	200 minutes	Lecture-based	↑ MIR score (p = 0.023), ↑ Satisfaction; OR failure = 5.61 (control group)
Shakeri, 2016 [14]	Iran	Cluster RTC	40	Software-supported teaching combined with a lecture	6 months	Lecture-based	↑ Knowledge and attitude (p < 0.05)
Sayed et al., 2018 [15]	Saudi Arabia	UPSS	180	PBL-integrated course	^*^	Pre-intervention vs. post-intervention	↑ Knowledge (p = 0.0001)
Wang et al., 2020 [16]	China	QE	88	Flipped classroom model	16 weeks	Lecture-based	↑ Interest, ↑ Performance (d ≈ 0.5)
Hayes et al., 2023 [17]	USA	UPPS	33	Interactive online module	^*^	Pre-intervention vs. post-intervention	↑ Knowledge and confidence (p < 0.001)
Bihari et al., 2021 [18]	India	RTC	96	PBL	^*^	Traditional lecture	↑ Total score (75.18 vs. 57.19; p = 0.01)
Bukumirić et al., 2022 [19]	Serbia	Pilot RTC	53	Hybrid PBL modules combined with blended learning	12 months	Blended learning without PBL	↑ Problem-solving skills (d = 0.69)
Chang et al., 2022 [20]	China	Cluster RTC	153	Seminar-case learning approach	108 hours	Lecture-based	↑ Total score and satisfaction (p < 0.05)
Evans et al., 2016 [21]	USA	QE^**^	279	Blended curriculum integrating case discussion, critical appraisal, clinical application, and teamwork (2013 cohort)	^*^	Traditional curriculum (2011–2012 cohorts)	↑ Satisfaction; no difference in exam scores
Freeman et al., 2008 [22]	UK	QE^**^	326	Interpretation-focused redesign (clinical contextualization, small tutorials, dramatized videos, animations)	10 hours	Traditional curriculum	↑ Conceptual understanding (p < 0.001)
Perry et al., 2014 [23]	Israel	UPPS	61	Intensive SPSS workshop with practical database simulation	30 hours	Pre-intervention vs. post-intervention	↑ Knowledge (p < 0.001)
Quinapanta et al., 2026 [24]	Ecuador	RTC	61	Algorithm-based multimodal method (algorithms, article analysis, SPSS simulation)	7 hours	Lecture-based	↑ Academic performance (p = 0.001)
Lancellotti et al., 2020 [25]	Chile	QE^**^	528	Team-based learning (structured collaborative active learning: independent preparation, formative assessment, collaborative learning) (2013–2016)	^*^	Lecture-based (2009–2011)	↑ High-complexity performance (p < 0.001)
Prihanti, 2017 [26]	Indonesia	QE	221	Biostatistics Center mentoring (research design, data collection and analysis, interpretation, follow-up)	7 weeks	No mentoring	↑ Competence (p < 0.001)
Tomak et al., 2026 [27]	Turkey	UPPS	234	Four-week biostatistics program (theoretical and practical sessions)	32 hours	Pre-intervention vs. post-intervention	↑ Knowledge across all domains (p < 0.001)

Academic Performance: Intervention vs. Control

**Figure 2:**
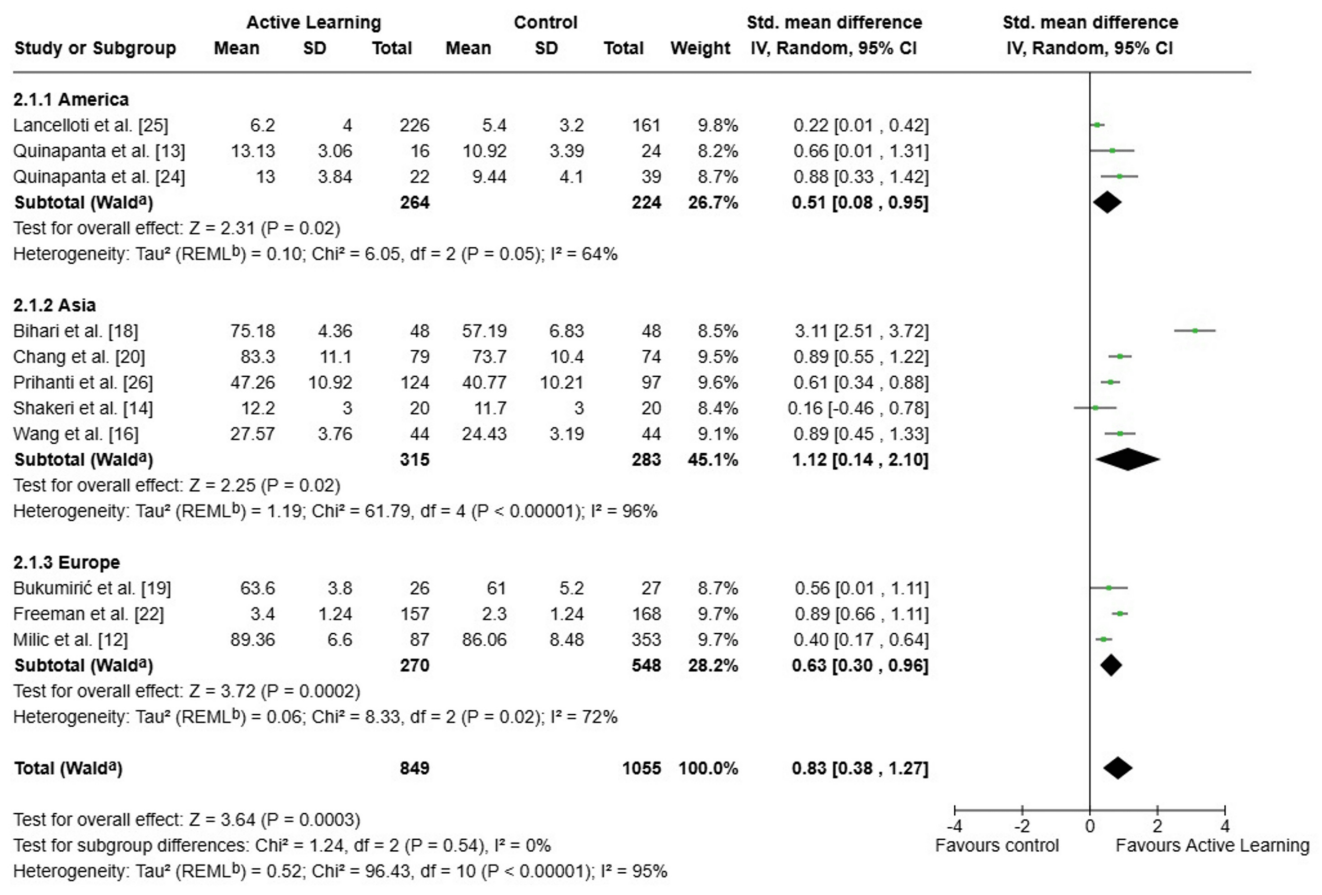
Forest plot: subgroup analysis of the effect of active methodologies on academic performance by continent ^a^CI calculated by the Wald-type method. ^b^Tau² calculated by the REML method (REML) SD: standard deviation; IV: inverse variance; CI: confidence interval; REML: restricted maximum-likelihood

Eleven studies analyzing the academic performance of students were included: 849 in the intervention group (active learning) and 1,055 in the control group (traditional methodology), as shown in Figure 2. Active learning was associated with a statistically significant improvement in performance compared to traditional teaching methods (SMD = 0.83; 95% CI: 0.38-1.27; p = 0.0003), corresponding to a large effect size. However, considerable heterogeneity was observed (I² = 95%), indicating substantial variability across studies. According to established benchmarks (Cohen's d > 0.8), a standardized mean difference of 0.83 falls within the "large" range, suggesting that replacing passive instruction with active methodologies may substantially improve students' mastery of biostatistics content. This is particularly relevant in medical education, where competency in biostatistics is essential for evidence-based clinical practice and the critical appraisal of scientific literature.

At the subgroup level, the effect was significant in the Americas (SMD = 0.51; 95% CI: 0.08-0.95; p = 0.02), Asia (SMD = 1.12; 95% CI: 0.14-2.10; p = 0.02) and Europe (SMD = 0.63; 95% CI: 0.30-0.96; p = 0.0002), as shown in Figure 2. This demonstrates the consistent benefits of the experimental group in different regional contexts, as well as showing homogeneity between regions (I² = 0%). The particularly pronounced effect observed in Asian studies (SMD = 1.12) may reflect contextual factors. In settings where traditional didactic instruction has historically predominated, introducing active, student-centred strategies may produce larger gains due to the novelty of the approach and students' relative unpreparedness to navigate complex statistical reasoning without scaffolded, participatory methodologies. 

**Figure 3:**
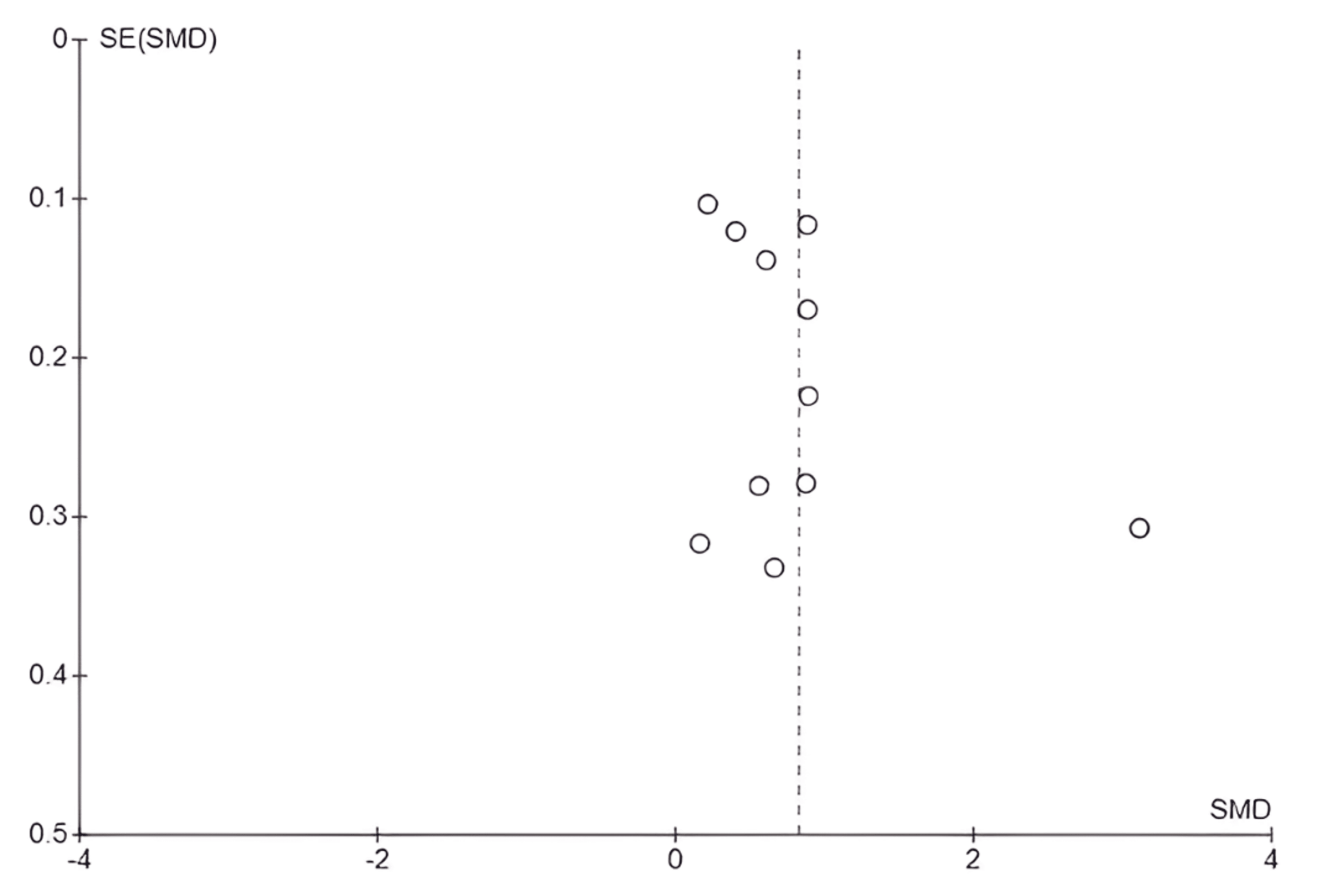
Funnel plot: effect of active methodologies on academic performance SE: standard error; SMD: standardized mean difference

The funnel plot shows slight asymmetry, with most studies close to the combined effect; however, one study had a greater effect and shifted to the right (Figure 3). However, Egger's test was not significant (t = 1.020; df = 9; p = 0.335), indicating that there is no statistical evidence of publication bias in the 11 included studies.

Sensitivity analysis: The sensitivity analysis confirmed the robustness of the results of the meta-analysis. Excluding the study by Bihari et al. [18], which was identified as an extreme outlier (DME = 3.11; 95% CI: 2.51-3.72), shifted the overall effect from a large effect size with very high heterogeneity (DME = 0.83; 95% CI: 0.38-1.27; p = 0.0003; I² = 95%) to a moderate effect size with considerably reduced heterogeneity (DME = 0.61; 95% CI: 0.42-0.80; p < 0.00001; I² = 66%) (see Appendices). This indicates that although this study influenced the magnitude and inconsistency of the overall estimate, excluding it does not reverse the direction or statistical significance of the effect favoring active learning. Additionally, the leave-one-out analysis confirmed that no individual study substantially alters the meta-analysis's conclusions, and the test for differences between subgroups was not significant (Chi² = 1.24; df = 2; p = 0.54; I² = 0%).

Pre-post Test Academic Performance

**Figure 4:**
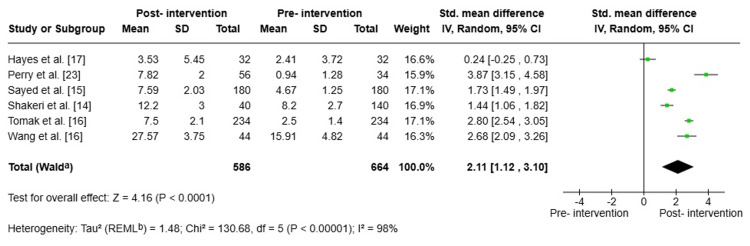
Forest plot: analysis of academic performance before and after active methodology implementation ^a^CI calculated by the Wald-type method. ^b^Tau² calculated by the REML method (REML) SD: standard deviation; IV: inverse variance; CI: confidence interval; REML: restricted maximum-likelihood

Across six studies involving 1,250 participants, the intervention was found to be associated with improved academic performance. This yielded a very large, statistically significant effect size (SMD = 2.11; 95% CI: 1.12-3.10; p < 0.0001), as illustrated in Figure 4. However, there was extreme heterogeneity (I² = 98%; τ² = 1.48; p < 0.00001), reflecting substantial variability between studies. This extreme heterogeneity probably stems from the wide diversity of interventions included, ranging from a seven-hour, algorithm-based, multimodal programme to a 32-hour, structured biostatistics course. This highlights the need for methodological caution when interpreting pooled pre-post estimates.

Sensitivity analysis: A sensitivity analysis using the leave-one-out method showed that the positive impact of active learning on academic performance is robust and remains statistically significant in all evaluated scenarios (p < 0.001), with an overall SMD ranging from 1.78 to 2.47, depending on which study was excluded. The two most influential studies were those of Hayes et al. [17] and Perry et al. [23]. Excluding the former increased the overall estimate (SMD: 2.47), and excluding the latter reduced it (SMD: 1.78). These studies acted as opposite ends of the spectrum. However, there was high heterogeneity among studies in all scenarios (I² = 94-98%), and although simultaneously excluding both studies reduced τ² from 1.48 to 0.42, this suggests that the observed variability reflected genuine differences in study design, population, and intervention, independent of the influence of individual studies.

Perception of Knowledge

**Figure 5:**
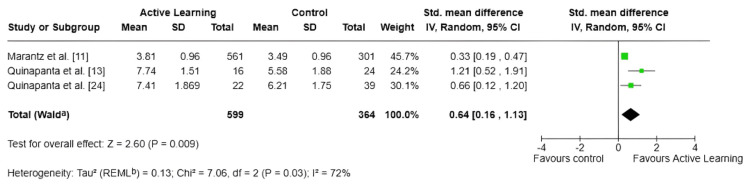
Forest plot: analysis of perceived knowledge ^a^CI calculated by the Wald-type method. ^b^Tau² calculated by the REML method (REML) SD: standard deviation; IV: inverse variance; CI: confidence interval; REML: restricted maximum-likelihood

As shown in Figure 5, three studies involving 963 medical students indicated that active learning was associated with a statistically significant improvement in knowledge retention compared with traditional teaching methods (SMD = 0.64; 95% CI: 0.16-1.13; p = 0.009), representing a moderate effect size. However, substantial heterogeneity was observed among the studies (I² = 72%).

Satisfaction With the Method

**Figure 6:**
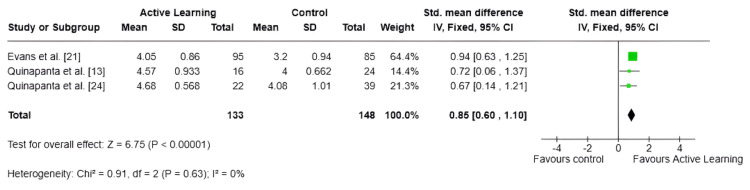
Forest plot: analysis of satisfaction ^a^CI calculated by the Wald-type method. ^b^Tau² calculated by the REML method (REML) SD: standard deviation; IV: inverse variance; CI: confidence interval; REML: restricted maximum-likelihood

Figure 6 shows that three studies involving 281 medical students demonstrated that active learning was associated with a statistically significant improvement in satisfaction compared to traditional teaching methods (SMD = 0.85; 95% CI: 0.60-1.10; p < 0.00001), representing a large effect size. There was no observed heterogeneity among the studies (I² = 0%; p = 0.63), indicating a high level of consistency across the findings.

Risk of Bias

**Figure 7:**
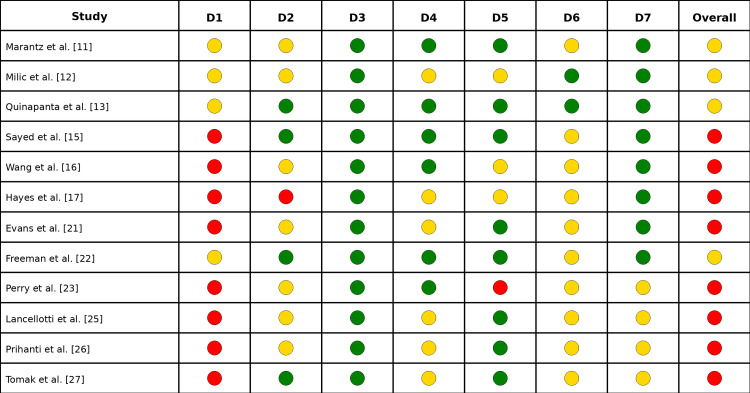
Risk of bias: ROBINS-I D1: confounding; D2: selection of participants; D3: classification of intervention; D4: deviations from intended intervention; D5: missing data; D6: measurement of outcomes; D7: selection of reported results. 🟢 Low risk. 🟡 Moderate risk. 🔴 Serious/critical risk ROBINS-I: Risk of Bias in Non-randomized Studies of Interventions

**Figure 8:**
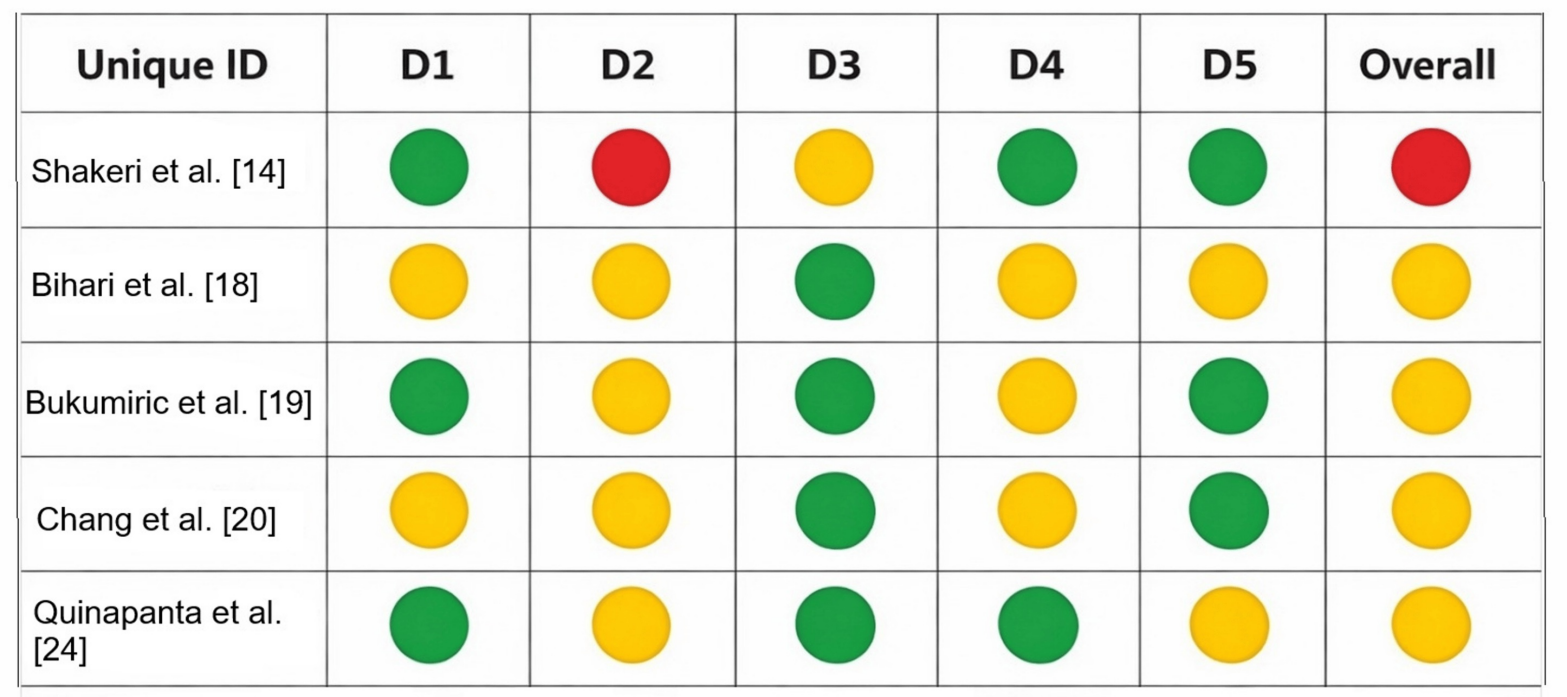
Risk of bias: RoB-2 D1: randomization process; D2: deviations from the intended interventions; D3: missing outcome data; D4: measurement of the outcome; D5: selection of the reported result. 🟢 Low risk. 🟡 Some concern. 🔴 High risk RoB-2: Risk of Bias 2.0

The ROBINS-I assessment showed a low risk of bias in the classification and application of the intervention, but a frequent serious risk of bias due to confounding factors, as well as a moderate risk of bias in the measurement and selection of outcomes (Figure 7). This resulted in an overall profile ranging from moderate to serious risk of bias, which requires caution. In RoB-2, low risk was observed in missing data and randomization in several cases (Figure 8). However, "some concerns" predominated in deviations, measurement, and selection of outcomes, indicating methodologically acceptable evidence with limitations requiring cautious interpretation.

Evidence Certainty (GRADE)

Using the GRADE framework, the overall evidence certainty ranged from very low to moderate across the four evaluated outcomes. Academic performance (vs. control) and perceived knowledge were both rated as having low certainty, primarily due to serious risk of bias from uncontrolled confounding factors and very high statistical heterogeneity. The pre-post academic performance outcome was rated as having very low certainty due to the absence of a control group and extreme heterogeneity (I² = 98%). Student satisfaction was rated as moderate certainty due to the absence of heterogeneity (I² = 0%) and a large, consistent effect across three studies. The complete Summary of findings is presented in the Appendices.

Discussion

This systematic review with meta-analysis synthesized evidence from 17 studies involving 3,702 undergraduate medical students from three continents to examine the effectiveness of active learning methods in teaching biostatistics in medical education. The main finding is that active learning strategies, including PBL, flipped classroom models, blended learning, case-based learning, and technology-supported instruction, produce significantly better results than traditional lecture-based teaching. The pooled effect size for academic performance was large and statistically significant (SMD = 0.83; 95% CI: 0.38-1.27; p = 0.0003), consistent with broader literature on active learning in health professions education. The magnitude of the observed effect is noteworthy. According to established benchmarks (Cohen's d > 0.8), a standardized mean difference of 0.83 falls within the "large" range, suggesting that replacing passive instruction with active methodologies could substantially improve students' mastery of biostatistics content.

However, the very high heterogeneity across the included studies (I² = 95%) warrants careful interpretation of the findings. This variability likely reflects genuine differences in the type, intensity, and duration of interventions; the academic level of participants; the outcome assessment instruments used; and the educational contexts in Asia, the Americas, and Europe. However, the subgroup analysis by continent revealed that the beneficial effect of active learning was consistent across all three regions (Americas: SMD = 0.51; Asia: SMD = 1.12; Europe: SMD = 0.63), with no statistically significant heterogeneity between subgroups (I² = 0%), which strengthens the generalizability of the conclusions.

The particularly pronounced effect observed in Asian studies (SMD = 1.12) may be due to contextual factors. In settings where traditional didactic instruction has historically predominated, introducing active, student-centred strategies may produce larger gains due to the novelty of the approach and students' relative unpreparedness to navigate complex statistical reasoning without scaffolded, participatory methodologies. This interpretation is supported by the findings of Yu et al. [28], whose meta-analysis of randomized controlled trials from Chinese medical institutions comprising 3,447 students demonstrated that PBL significantly outperformed LBL across all evaluated outcomes in health statistics courses. The effect sizes reported for learning interest (RR = 2.24; 95% CI: 1.15-4.38) and self-learning ability (RR = 1.99; 95% CI: 1.48-2.67) were particularly large. This suggests that PBL may have a greater impact on motivational and metacognitive dimensions in contexts where rote learning has traditionally been used.

The pre-post analysis further reinforces these conclusions. Among six studies involving 1,250 participants, the pooled SMD was 2.11 (95% CI: 1.12-3.10; p < 0.0001), representing a very large within-group effect size. Although uncontrolled pre-post designs are susceptible to threats to internal validity, including maturation effects, regression to the mean, and test-retest practice effects, the magnitude of the observed gains across diverse interventions and settings suggests a genuine learning effect, which is at least partly attributable to the active components of the instruction. The extreme heterogeneity in this analysis (I² = 98%) was likely driven by differences in methodology, population, and implementation.

Active learning was associated with significant improvements in both academic performance and perceived knowledge (SMD = 0.64; 95% CI: 0.16-1.13; p = 0.009), as well as student satisfaction (SMD = 0.85; 95% CI: 0.60-1.10; p < 0.00001). The latter finding is particularly robust given the absence of heterogeneity across the three contributing studies (I² = 0%; p = 0.63). High satisfaction scores associated with active learning methods have been consistently reported in related fields and are not merely of peripheral importance; student satisfaction influences engagement, motivation, and the long-term retention of knowledge. Furthermore, an enhanced sense of perceived knowledge (the subjective feeling of competence and mastery) can reduce statistical anxiety, which remains a significant barrier to effectively acquiring biostatistics among medical students.

One of the most plausible mechanisms underlying these satisfaction gains is the reduction in statistical anxiety. Ordak [29] directly examined this dimension by surveying 527 students across seven medical faculties at nine universities in Poland. In the practical form, students first analysed a published article in which the test in question was applied, and then performed the analysis using a step-by-step guide. In all seven fields of study and in the pooled group, the practical approach was associated with significantly lower class-related stress, lower examination stress, greater perceived practical knowledge acquisition, and higher post-examination satisfaction. Importantly, the traditional approach was also associated with a significantly greater perceived difficulty in interpreting published research results in the future (p < 0.001), a finding with direct implications for clinical competence and evidence-based practice. Yu et al. [28] expanded on this by showing that PBL also leads to measurable improvements in non-cognitive outcomes, specifically collaboration skills (RR = 1.25; 95% CI: 1.09-1.45) in statistics. This dimension is not sufficiently captured by the outcome framework of the present review, but is highly relevant to the development of professional competency in medicine.

These findings align with and extend those of previous reviews in this field. In their landmark meta-analysis of active learning in undergraduate STEM education, Freeman et al. [30] reported an average SMD of 0.47 for examination scores and a 1.5-fold increase in failure rates in traditional courses compared to active learning courses. The larger effect size observed in the present review (SMD = 0.83) may be partly explained by the motivational significance of applying statistical methods to medical content, the high level of academic commitment generally exhibited by medical students, and the clinical relevance of biostatistics in professional practice. Importantly, this argument is supported by a convergent body of diagnostic evidence from three independent cultural settings. In China, for example, Chen et al. [31] found that almost half (48%) of the 502 medical students surveyed considered the current teaching approaches to be inappropriate, with only 22.5% expressing a positive attitude towards formula derivation.

In Saudi Arabia, Sayed et al. [32] argued that statistics anxiety constitutes a structural barrier that can only be overcome by repositioning statistics as a research tool rather than an examinable subject. In Poland, Ordak [29] reported that traditional teaching was associated with substantially higher stress, with median class-related stress scores of 65-80 (out of 100), compared to 20-30 for practical teaching. A statistically significant majority of students (93-96% across all fields) believed that university staff should be required to attend additional biostatistics training. This finding implicitly indicates the inadequacy of current instructional approaches. Taken together, this multi-continental convergence of student dissatisfaction constitutes a powerful driver of demand for the adoption of active learning and suggests that the effect sizes observed in intervention studies may partly reflect relief from aversion rather than mere instructional efficiency gains.

From a methodological standpoint, important limitations are evident in the quality of the evidence base. A risk of bias assessment using ROBINS-I revealed that non-randomized studies, which constitute the majority of the included evidence, frequently exhibited a serious risk of bias due to uncontrolled confounding factors. Historical cohort comparisons, in which cohorts taught under different curricula are compared, are particularly susceptible to secular trends in student performance and changes in curriculum content unrelated to the pedagogical approach under evaluation. The RoB-2 assessment of randomized trials showed that, although the randomization process and missing data were generally satisfactory, several studies had concerns regarding deviations from intended interventions and measurement bias. These concerns echo those of Yu et al. [28], who, despite classifying all included studies as randomized controlled trials, acknowledged persistent selection and performance bias. Evidence suggests that active methodologies are an effective strategy for enhancing academic performance, knowledge retention, and satisfaction with the teaching of biostatistics among medical students, thereby supporting their integration into medical education curricula.

Limitations

Although the results show positive effects of active learning compared to traditional teaching, there are important limitations that should be acknowledged. Heterogeneity between studies was very high, reflecting differences in contexts, samples, and types of intervention, which limits generalizability. While the effects were statistically significant (p < 0.05) and there was no evidence of publication bias, the overall risk of bias was moderate to serious in several studies, meaning the findings should be interpreted with caution.

Future research directions

Future research should focus on conducting well-designed randomized controlled trials with standardized outcome measures, in order to reduce heterogeneity and improve the certainty of the evidence base. Long-term follow-up studies would also be valuable in determining whether the benefits of active learning in biostatistics lead to improved research literacy and evidence-based practice among future physicians.

## Conclusions

This systematic review with meta-analysis indicates that active learning methodologies are more effective than traditional methods for teaching biostatistics to undergraduate medical students. Compared to lectures, active learning strategies were found to significantly improve academic performance, with high effect sizes suggesting a deeper understanding of statistical content. Active learning was also associated with a greater perception of knowledge, greater satisfaction with the teaching process, and a more motivating, participatory educational experience. However, these findings should be interpreted with caution due to high heterogeneity among the included studies, as well as a moderate to serious risk of bias in some of them. These methodological limitations underscore the necessity for future research employing more rigorous and standardized designs to corroborate and reinforce the evidence regarding the efficacy of active methodologies in biostatistics education.

## Figures and Tables

**Figure 9:**
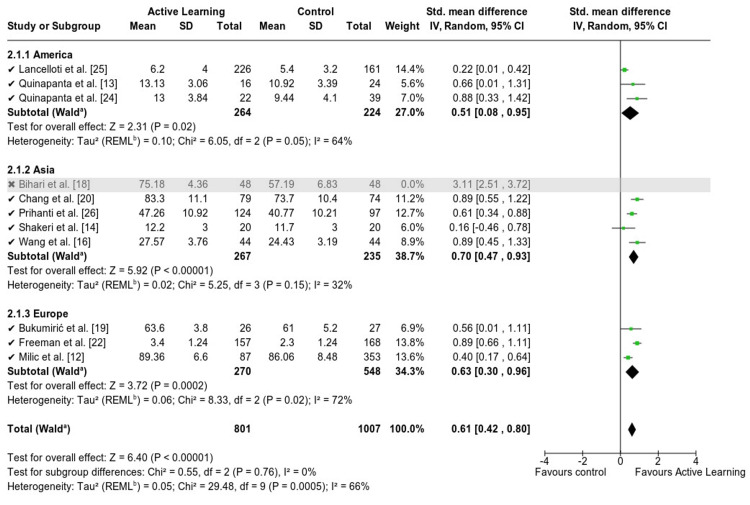
Sensitivity analysis ^a^CI calculated by the Wald-type method. ^b^Tau² calculated by the REML method (REML) SD: standard deviation; IV: inverse variance; CI: confidence interval; REML: restricted maximum-likelihood

## Appendices

**Table 3:** GRADE summary of findings: certainty of evidence GRADE scale: ⊕⊕⊕⊕ High; ⊕⊕⊕◯ Moderate; ⊕⊕◯◯ Low; ⊕◯◯◯ Very low ᵃRisk of bias: serious confounding in non-randomized studies (ROBINS-I); some concerns in RCTs (ROB 2.0). Downgraded: –1 to –2 ᵇInconsistency: I² = 72–98%; variability in interventions, populations, and instruments. Between-subgroup I² = 0%. Downgraded: –1 to –2 ᶜIndirectness: population and setting directly match the review question. Not downgraded ᵈPublication bias: Egger's test non-significant (p = 0.335). Not downgraded ᵉPublication bias (pre-post): not assessable in uncontrolled designs. Not upgraded or downgraded ᶠImprecision: only 3 studies (n = 963); wide 95% CI. Optimal information size not met. Downgraded: –1 ᵍSatisfaction homogeneity: I² = 0% (p = 0.63); high consistency across 3 studies GRADE: Grading of Recommendations, Assessment, Development and Evaluation; SMD: standardized mean difference; CI: confidence interval; I²: heterogeneity statistic; RCT: randomized controlled trial; PBL: problem-based learning

Outcome	No. of studies	No. of participants	Risk of bias	Inconsistency	Indirectness	Imprecision	Publication bias	Effect size SMD (95% CI)	I²	Certainty (GRADE)
Academic performance (active vs. traditional)	11	1,904 (849 vs. 1,055)	Seriousᵃ	Very seriousᵇ (I² = 95%)	Not seriousᶜ	Not serious	Undetectedᵈ	SMD = 0.83 (0.38–1.27) p = 0.0003	95%	⊕⊕◯◯ Low
Academic performance (pre–post analysis)	6	1,250	Very seriousᵃ	Very seriousᵇ (I² = 98%)	Not seriousᶜ	Not serious	Not assessedᵉ	SMD = 2.11 (1.12–3.10) p < 0.0001	98%	⊕◯◯◯ Very low
Perceived knowledge (self-reported)	3	963	Seriousᵃ	Seriousᵇ (I² = 72%)	Not seriousᶜ	Seriousᶠ	Not assessed	SMD = 0.64 (0.16–1.13) p = 0.009	72%	⊕⊕◯◯ Low
Student satisfaction with the teaching method	3	281	Seriousᵃ	Not seriousᵍ (I² = 0%)	Not seriousᶜ	Not serious	Not assessed	SMD = 0.85 (0.60–1.10) p < 0.00001	0%	⊕⊕⊕◯ Moderate
